# Correlation analysis of serum cytokine levels and immune inflammatory factors in cirrhotic portal hypertension

**DOI:** 10.5937/jomb0-60996

**Published:** 2026-04-15

**Authors:** Yafeng Yuq, Zhiqiong Lan, Zhiren Huang, Lingqin Hu

**Affiliations:** 1 Affiliated Hospital of Shaoxing University (Shaoxing Municipal Hospital), Department of Infectious Diseases (Hepatology), Shaoxing City, China; 2 Jiangsu Provincial People's Hospital, Department of Gastrointestinal Surgery, Nanjing, China; 3 Affiliated Hospital of Shaoxing University (Shaoxing Municipal Hospital), Department of Geriatrics, Shaoxing City, Zhejiang Province, China

**Keywords:** liver cirrhosis, portal hypertension, immune-inflammatory factors, cytokine levels, ciroza jetre, portalna hipertenzija, imunoinflamatorni faktori, nivoi citokina

## Abstract

**Background:**

To investigate alterations in immune-inflammatory factors and variables, including procalcitonin(PCT), prostaglandin E (PGE), and serum C-reactive protein (CRP) in individuals with cirrhotic portal hypertension.

**Methods:**

From March 2020 to March 2025, 81 patientswith cirrhotic portal hypertension and 50 healthy volunteerswere hospitalised to our hospital. These people were divided into two groups: the portal hypertension group and thecontrol group. Aspartate aminotransferase (AST), albumin(ALB), total bilirubin (TBIL), alanine aminotransferase(ALT), and portal vein inner diameter (Pvd) were measured.Serum cytokines, including CRP PCT, and PGE, weremeasured, as well as the percentages of CD 4+/CD 8+ Tcells and CD 3+ , CD 4+ , and CD8+ T lymphocytes.

**Results:**

The group with portal hypertension had considerablylower ALB than the control group (P&lt; 0.05), whereas indicators including ALT, AST, TBIL, and Dpv were significantlyhigher in the portal hypertension group than in the controlgroup (P&lt; 0.05). While cytokine markers, including CRPPCT, and PGE, were higher in the portal hypertension groupthan in the control group (P&lt; 0.05), the numbers of CD3 +and CD4+ T lymphocyte subsets and CD4+/CD8+ T lymphocytes were considerably lower in the portal hypertensiongroup. With increasing liver function classification, the proportions of CD3+ and CD4+ T lymphocyte subsets, as wellas CD4+/CD 8+ T lymphocytes, decreased in patients. However, there was a statistically significant (P&lt;0.05) increase in the levels of serum PCT, PGE, and CRP Spearmancorrelation analysis revealed that liver function classificationwas negatively correlated with immune indicators. CRP PCTand PGE levels were favourably connected with CD4+ andCD4+/CD 8+ values (P &lt; 0.05). Dpv was shown to have anegative association with CD4+/CD8+ T cells and a positive correlation with PCT and PGE levels (P&lt;0.05) accordingto Pearson correlation analysis.

**Conclusions:**

Patients with cirrhotic portal hypertensionmay experience immune dysfunction and cytokine disorders. These changes are related to liver function and portalvein pressure and can be used as reference indicators forevaluating a patient's condition in clinical practice.

## Abstract (sr)

**Uvod:**

Cilj je bio da se ispitaju promene imunoinflamatornih faktora i parametara, uključujud prokalcitonin(PCT), prostaglandin E (PGE) i serumski C-reaktivni protein (CRP), kod bolesnika sa cirotičnom portalnomhipertenzijom.

**Metode:**

U periodu od marta 2020. do marta 2025. go -dine, u našoj ustanovi hospitalizovan je 81 pacijent sa cirotičnom portalnom hipertenzijom i 50 zdravih dobrovo Ijaca.Ispitanici su podeljeni u dve grupe: grupu sa portalnomhipertenzijom i kontrolnu grupu. Mereni su aspartat-aminotransferaza (AST), albumin (ALB), ukupni bilirubin (TBIL),alanin-aminotransferaza (ALT) i unutrašnji dijametar portalne vene (Pvd). Određivani su serumski citokini, uključujuciCRP PCT i PGE, kao i procenat CD 4+/CD 8+ T celija isubpopulacija CD 3+, CD4+ i CD8+ T limfocita.

**Rezultati:**

Grupa sa portalnom hipertenzijom imala jeznačajno niže vrednosti ALB u poređenju sa kontrolnomgrupom (P &lt; 0 ,0 5), dok su ALT, AST, TBIL i Pvd bili značajno viši (P &lt; 0,05). Citokinski markeri (CRP PCT i PGE)su bili povišeni u grupi sa portalnom hipertenzijom(P &lt; 0,05). Procenat CD3+ i CD4+ T limfocita, kao iodnos C D 4+ /C D 8+ , su bili značajno niži u odnosu nakontrolnu grupu. Sa pogoršanjem klasifikacije funkcijejetre, procenat CD3+ i CD4+ T limfocita i odnosCD 4+ /CD 8+ dalje su se smanjivali, dok su vrednosti PCT,PGE i CRP značajno rasle (P &lt; 0,05). Spearmanova korelaciona analiza pokazala je negativnu povezanost klasifikacij'e funkcje j'etre sa imunološkim parametrima. Nivoi CRPPCT i PGE su bili pozitivno korelisani sa vrednostimaCD4+ i CD 4+ /CD 8+ (P &lt; 0,05). Pearsonova analizapokazala je da je Pvd negativno povezan sa CD 4+/CD 8 +T celijama, a pozitivno sa nivoima PCT i PGE (P&lt; 0,05).

**Zaključak:**

Bolesnici sa cirotičnom portalnom hipertenzijom pokazuju imunsku disfunkciju i poremecaje citokinskog profila. Ove promene su u vezi sa funkcijom jetre i pritiskom u portnoj veni i mogu se koristiti kao referentniparametri za kliničku procenu stanja bolesnika.

## Introduction

One major cause of the elevated patient death rate in patients with liver cirrhosis is portal hypertension, a dangerous consequence. The blood circulation of the liver is rather unique, featuring dual blood supplies from the hepatic artery and portal vein. Both finally join the inferior vena cava through the hepatic vein after passing through the hepatic sinusoids. Therefore, when the pressure in the hepatic sinusoids increases, portal hypertension is very likely to occur. Additionally, an increase in the resistance of the hepatic sinusoids can also cause the blood in the hepatic artery to flow through the peripheral vessels to the portal vein, further increasing the pressure in the portal vein [1][2]. The production of inflammatory factors in the liver due to various circumstances is the pathological underpinning of liver cirrhosis, promoting collagen synthesis and the activation of hepatic stellate cells. Collagen substances cover the surface of the vascular endothelium, causing capillarisation, which makes it difficult for blood flow in the hepatic sinusoids to supply nutrients to the liver tissue through the micropores between endothelial cells, thereby leading to extensive necrosis of liver cells. Moreover, the deposition of a large amount of collagen in the Disse space can also cause the hepatic sinusoids to narrow, leading to blocked blood flow and, consequently, portal hypertension [3]. Numerous studies [4][5] have confirmed that patients with liver cirrhosis often exhibit changes in immune function and increased serum levels of inflammatory cytokines. However, there are still no reports on the relationships between these changes and liver function or portal hypertension.

The proportion of T lymphocyte subsets and the levels of inflammatory indicators like procalcitonin (PCT), C-reactive protein (CRP), and prostaglandin E (PGE) were used as research indicators in this study to examine changes in each indicator in patients with cirrhotic portal hypertension and their relationships to portal vein pressure and liver function. To provide a reference basis for the assessment of the condition of patients with cirrhotic portal hypertension.

## Materials and methods

### General information

From March 2020 to March 2025, 81 patients with cirrhotic portal hypertension who were hospitalised to our hospital were chosen as research participants and placed in the portal hypertension group. Inclusion criteria: aged 18 to 65 years and had a positive serum hepatitis B virus antigen marker test for more than 6 months. Patients who met the diagnostic criteria for portal hypertension, as outlined in the Bayraktar criteria [6][7], were voluntarily enrolled. Exclusion criteria: coinfection with hepatitis C, hepatitis D or HIV; hepatocellular carcinoma confirmed by pathology; chronic diseases such as diabetes, hypertension and coronary heart disease; mental disorders; and current use of hormones or immunosuppressants. The control group consisted of 50 healthy volunteers who underwent physical examinations at our hospital. The subjects in the control group underwent comprehensive physical examinations and were excluded if they had chronic diseases, immune diseases, liver diseases, or other relevant conditions.

This study has been approved by the Medical Research Ethics Committee (GyZL-ZN-2020-045).

### Research methods

Venous blood was collected from all the enrolled subjects, and aspartate aminotransferase (AST), alanine aminotransferase (ALT), and total bilirubin levels were determined via an automatic biochemical analyser. Liver function indicators such as bilirubin (TBIL) and albumin (ALB) were measured. Colour Doppler ultrasound was used for examination to collect data on portal vein diameter (PVD). The T lymphocyte subsets of the patients were classified by flow cytometry, and the levels of CD4+/CD8+ T cells, as well as the counts of CD3+, CD4+, and CD8+ T lymphocytes, were measured. PGE, PCT, and serum CRP were measured using an enzyme-linked immunosorbent test.

### Laboratory biochemical testing methods

5 mL of venous blood was collected on an empty stomach in the early morning. After coagulation at room temperature for 30 minutes, the sample was centrifuged at 3000 g for 10 minutes at 4°C. The serum was then aliquoted and stored at -80°C, avoiding repeated freezing and thawing.

Cytokines (IL-6, IL-8, TNF-α, IL-1β, TGF-β1) were treated by magnetic bead multi-factor method: Bio -PLEX 200 system (Bio -rad, USA), with Bio -plex Pro Human Cytokine Panel (Bio -rad, item number with selected panel, example M5000121, check before purchase); If ELISA is used instead, the R&D Systems Quantikine kit, sample item number: Il-6 D6050, TNF-α DTA00D, IL-8 D8000C, IL-1β DLB50, TGF-β1 DB100B (all are examples, check according to the batch manual), 450 nm reading. Immune inflammatory factor: hs-CRP was detected by immunoturbidimetry in Cobas c702 (Roche Diagnostics, Germany); C3/C4 and IgG/IgA/IgM were measured by scattering immunoturbidimetry in BN II (Siemens Healthineers, Germany), all using the original factory-matched reagents.

### Measurement methods for enzymes and other biomarkers

Five millilitres of fasting venous blood were collected from the research subjects. After standing at room temperature for 30 minutes, it was centrifuged at 3000 rpm for 15 minutes (Centrifuge: Eppendorf Centrifuge 5804R, Germany). Separate the serum, aliquot it into sterile EP tubes, and freeze it in a Thermo Scientific Forma 900 series (USA) at -80°C for testing. Avoid repeated freezing and thawing. Thaw and mix evenly before testing.

The following key factors in serum were quantitatively detected by Enzyme-Linked Immunosorbent Assay (ELISA):

Pro-inflammatory cytokines: interleukin-6 (IL-6), tumour necrosis factor-α (TNF-α), interleukin-1β (IL-1β); Anti-inflammatory cytokine: Interleukin-10 (IL-10) Chemokine: Interleukin-8 (IL-8/CXCL8); Acute-phase protein: C-reactive protein (CRP); Fibrosome-related factors: Transforming growth factor-β1 (TGF-β1), vascular endothelial growth factor (VEGF).

Fully automatic microplate reader (BioTek Synergy H1 multifunctional microplate detector, USA). Use the human-specific ELISA kit (Manufacturer: R&D Systems, USA; sample item number: IL-6: D6050) and operate strictly in accordance with the instructions. The experiment employed the double antibody sandwich method, and all steps were performed at room temperature and in a dark environment.

### Statistical analysis

Data processing and statistical analysis were conducted using SPSS 20.0. Count data is expressed using [n(%)]. Fisher's exact test, often known as the χ^2^ test, was used to compare different groups. Count data conforming to a normal distribution are expressed as the mean ± standard deviation (x̄ ± s). One-way ANOVA was used for comparisons among multiple groups, and independent sample t-tests were conducted for comparisons between groups. Pearson correlation analysis or Spearman correlation analysis was used for the correlations among indicators.

## Results

### Comparison of liver function-related indicators

While the portal hypertension group had significantly higher levels of indicators, including ALT, AST, TBIL, and Pvd, than the control group (P<0.05), the portal hypertension group also had significantly lower levels of the ALB index (P<0.05) than the control group (Table 1).

**Table 1:** Comparison of liver function and portal vein pressure-related indicators among subjects in each group.

Group	Number of cases	ALT (U/L)	AST (U/L)	TBIL (μmol/L)	ALB (g/L)	Pvd (mm)
Portal hypertension<br>group	81	44.15±10.23	33.01±8.25	15.01±4.22	40.18±8.56	14.01±2.85
Control group	50	18.56±6.82	22.14±7.01	10.17±2.56	45.96±7.85	10.23±2.01
t value	-	15.659	7.746	7.315	3.873	8.199
P value	-	<0.001	<0.001	<0.001	<0.001	<0.001

### Immune-inflammatory factors and cytokine levels

The portal hypertension group had significantly higher serum levels of PCT, PGE, and CRP than the control group (P<0.05). Additionally, the proportions of CD3+ and CD4+ T lymphocyte subsets, as well as CD4+/CD8+ T lymphocytes, were significantly lower (P<0.05) (Table 2).

**Table 2:** Comparison of immune-inflammatory factors among subjects in each group.

Group	Number<br>of cases	CD3+(%)	CD4+(%)	CD8+(%)	CD4+/CD8+	CRP<br>(mg/L)	PCT<br>(ng/mL)	PGE<br>(pg/mL)
Portal hypertension<br>group	81	68.45±7.85	35.45±5.56	36.15±6.23	0.97±0.25	30.25±12.89	5.89±2.11	1527.45±452.26
Control group	50	78.25±7.22	47.18±5.56	36.82±5.82	1.25±0.52	5.14±1.22	0.33±0.10	623.52±127.48
t value	-	7.154	11.730	0.613	4.139	13.717	18.592	13.781
P value	-	<0.001	<0.001	0.541	0.001	<0.001	<0.001	<0.001

### Cytokine levels and immune-inflammatory factors markers in patients with varying liver function grades

Patients with varying liver function grades exhibited statistically significant differences in cytokine levels and inflammatory immune factors.

With increasing liver function grade, the proportions of CD3+ and CD4+ T lymphocyte subsets, as well as CD4+/CD8+ T lymphocytes, in patients decreased, while the levels of serum cytokines, such as CRP, PCT, and PGE, increased (Table 3).

**Table 3:** Comparison of inflammatory mediator Levels among subjects in each group.

Indicator	Number<br>of cases	CD3+(%)	CD4+(%)	CD8+(%)	CD4+/CD8	CRP (mg/L)	PCT (ng/mL)	PGE (pg/mL)
Child Grade<br>A	25	70.15±7.01	37.14±5.12	35.45±4.17	1.12±0.27	22.41±6.82	5.01±1.01	1302.41±389.52
Child Grade<br>B	30	66.01±7.14	34.15±4.11	36.12±5.01	0.90±0.29	29.86±7.15	5.82±1.34	1574.15±428.56
Child Grade<br>C	26	63.23±6.56	30.12±5.26	37.14±4.18	0.76±0.30	34.17±8.22	6.45±0.98	1785.45±485.62
F value	-	6.449	13.669	0.914	10.157	16.395	10.291	7.835

### Correlation analysis of immune-inflammatory factors and inflammatory mediators with liver function classification and portal vein pressure

The classification of liver function was found to be positively correlated with CRP PCT, and PGE levels (P<0.05) and negatively correlated with immunological indicators, such as CD4+ and CD4+/CD8+, according to Spearman correlation analysis. Pvd was found to be favourably associated with PCT and PGE levels (P<0.05) (Table 4).

**Table 4:** Correlation analysis of immune-inflammatory factors, inflammatory mediators, with liver function classification and portal vein pressure.

Indicator	Classification of liver function		Pvd	
	r value	P value	r value	P value
CD3 +	-0.124	0.159	-0.222	0.236
CD4 +	-0.356	0.014	-0.274	0.156
CD8 +	0.174	0.345	0.214	0.356
CD4+/CD8	-0.401	0.001	-0.389	0.047
CRP	0.315	0.035	0.102	0.052
PCT	0.347	0.041	0.352	0.028
PGE	0.301	0.042	0.326	0.034

## Discussion

When portal hypertension in liver cirrhosis occurs and progresses, the cellular immune function of the spleen may be disrupted to varying degrees, mainly in a state of low immune function dominated by cellular immunity [8][9][10][11]. Current research suggests that the leading causes of hyperfunction of the spleen when portal vein cirrhosis occurs are related to the following aspects: portal hypertension leads to a highly dynamic state of blood flow in the spleen and other internal organs, increasing lateral vascular pressure, damaging the vascular endothelium, and causing damage to the spleen vessels; under the effect of portal hypertension, promoting the proliferation of the vascular endothelium and increasing vascular permeability [12][13][14]. The combination of these two factors leads to the proliferation of splenic vessels and an increase in splenic pressure, causing hypersplenism and aggravating the state of portal hypertension [15]. When cirrhotic portal hypertension occurs, the splenic sinuses dilate and become congested [16]. Uncontrolled passive congestion over an extended period may cause fibrous tissue to proliferate and endothelial cells in the sinus wall, resulting in basement membrane hyperplasia of the splenic sinus wall, thickening of the splenic cords, and an increase in fibrous tissue, leading to complications such as splenomegaly [17][18][19][20].

As a peripheral immune organ, the spleen is the site where the body receives antigens and generates specific immune responses [21][22][23]. In portal hypertension, the spleen becomes congested and enlarged due to tissue hypoxia, leading to modifications in immune cell architecture and function, which in turn compromise the body's immunological function [24][25][26]. The results of this study show that, compared with normal adults, patients with cirrhotic portal hypertension may have an imbalance in the proportion of T lymphocyte subsets, and the imbalance increases with increasing liver function rating. Correlation analysis also revealed that liver function classification, Pvd and immune indicators such as CD4+ and CD4+/CD8+ are negatively correlated to a certain extent, suggesting that the proportion of T lymphocyte subsets can, to a certain extent, reflect the occurrence and development of the condition in patients with cirrhotic portal hypertension [27][28][29]. There have been few previous reports on the immune function of patients with cirrhotic portal hypertension. This study further confirmed that portal hypertension in patients with liver cirrhosis can lead to immune dysfunction and that the patient's condition is intimately linked to the degree of this malfunction.

In addition to immune conditions, inflammatory factors also play a significant role in the occurrence of portal vein cirrhosis [30][31][32][33]. Various immunological responses in the body are mediated by inflammatory factors, which are polypeptide compounds produced by immune cells that have different biological consequences. As an inflammation-mediated disease, liver cirrhosis has been reported by numerous studies to be prone to hypersynthesis of inflammatory factors [34]. CRP PCT and PGE in patients with portal hypertension are elevated, and the classification of liver function and portal vein pressure is connected with these levels. Liver cells release the acute-phase protein known as CRP which is a sensitive indicator of inflammation and tissue damage. PCT is a propeptide substance of serum calcitonin and is overexpressed in infectious diseases. Previous studies [35][36][37] have shown that abnormal liver function in patients with liver cirrhosis can stimulate the synthesis and secretion of PCT in liver macrophages and monocytes. In addition, the disorder of the intestinal flora in patients with liver cirrhosis may also be an important reason for the increased synthesis of PCT [38]. PGE is an immunosuppressive factor that regulates immune function and inhibits the secretion of cytokines, etc.

## Conclusion

Patients with cirrhotic portal hypertension may present with immune dysfunction and cytokine disorders. This change is related to the patient's liver function and portal vein pressure and can be used as a reference indicator for evaluating the patient's condition in clinical practice.

## Dodatak

### Acknowledgements

We thank all patients for participating in this study.

### Funding

None.

### Conflict of interest statement

All the authors declare that they have no conflict of interest in this work.
